# Optimal Structure of a Plasmonic Chip for Sensitive Bio-Detection with the Grating-Coupled Surface Plasmon-Field Enhanced Fluorescence (GC-SPF)

**DOI:** 10.3390/ma10091063

**Published:** 2017-09-11

**Authors:** Keiko Tawa, Takuya Nakayama, Kenji Kintaka

**Affiliations:** 1 School of Science and Technology, Kwansei Gakuin University, 2-1 Gakuen, Sanda, Hyogo 669-1337, Japan; taku2009.2.27@ezweb.ne.jp; 2 Inorganic Functional Materials Research Institute, National Institute of Advanced Industrial Science and Technology (AIST), 1-8-31 Midorigaoka, Ikeda, Osaka 563-8577, Japan; kintaka.kenji@aist.go.jp

**Keywords:** plasmon, fluorescence, grating-coupled SPR, silver-film thickness, SPCE, sensitive detection, fluorescence enhancement factor

## Abstract

Surface plasmon field-enhanced fluorescence (SPF) has been one of the powerful tools for biosensors and bioimaging. A wavelength-scale periodic structure coated with a thin metal film is called a plasmonic chip, and it can provide SPF. SPF of Cy5-streptavidin (Cy5-SA) was measured on a biotinylated plasmonic chip with a grating of 480 nm-pitch. The optimal structure of a plasmonic sensor-chip was designed for improving detection sensitivity. The silver film thickness dependence of the SPF intensity was measured under the irradiation of the top panel of a sensor chip. Furthermore, the dependence of the SPF intensity on the distance from the metal surface was also investigated. The optimal structure for the largest fluorescence enhancement factor was 150 nm-thick silver and 10 nm-thick SiO_2_ layers due to the enhanced electric field (excitation field), the surface plasmon coupled emission (SPCE), and the interference effect with reflected light. The largest enhancement factor was found to be 170-fold. Furthermore, not only the largest fluorescence intensity but also stable lower background noise were found to be essential for higher-sensitive detection.

## 1. Introduction

Surface plasmon field-enhanced fluorescence (SPF) has been known as one of the powerful tools in immunosensors [1,2]. This is because fluorescence is a sensitive technique compared with methods measuring absorbance and reflectivity, and further, enhanced fluorescence can improve the detection sensitivity compared with the general fluorescence method. In SPF, the surface plasmon resonance (SPR) field is utilized as an excited field for fluorescent molecules. SPR is classified to propagating SPR and localized SPR. Localized SPR has been shown in nanoparticle-arrays and nanostructure-arrays with a size of several nm to several tens of nm [3,4]. Recently, localized SPR has been mainly studied for surface-enhanced Raman scattering (SERS) spectroscopy [3,5], photochemical reaction [6,7], light harvesting [8], and photonics [9,10]. It has the advantage of strongly enhanced electric field, but the area of the enhanced field is quite limited. On the other hand, localized SPR and propagating SPR have also been applied to SPF [11,12,13]. In fluorescence immunosensors, a large area is generally required for a sensor chip. So, propagating-SPF has an advantage due to the fabrication of a regular nano-structure array in a wide region and the less strict control of a distance from a metal surface compared with a localized-SPF. Basically, the fluorescence enhancement can be interpreted from the improvement of fluorescence quantum efficiency in Jablonski diagrams [14]. The fluorescence quantum efficiency *η* is given by *η* = k_r_/(k_r_ + k_nr_) using radiative and nonradiative decay rate constants, respectively. When the molecule is located on the plasmonic chip, energy transfer to surface plasmons with a rate k_sp_’ and interband or intraband transitions with a rate k_isw_’ were added, and fluorescence quantum efficiency was modified to *η*’= k_r_’/(k_r_’ + k_nr_ + k_sp_’ + k_isw_’). Moreover, when the possibility for the plasmon to reradiate is added as the probability χ = Г_r_/(Г_r_ + Г_a_), where Г_r_ and Г_a_ are the radiative decay rate and the absorption decay rate, respectively, *η*’ is given by

*η*’ = (k_r_’ + χk_sp_’)/(k_r_’ + k_nr_ + k_sp_’ + k_isw_’) (1)

The enhancement factor for quantum efficiency is

*η*’/*η* = (k_r_’ + χk_sp_’) (k_r_ + k_nr_)/k_r_ (k_r_’ + k_nr_ + k_sp_’ + k_isw_’) (2)

The fluorescence enhancement based on the excitation process is found to be composed of the increase of quantum efficiency and the enhanced excited-field.

In propagating SPR, there are prism-coupled SPR (PC-SPR) and grating-coupled SPR (GC-SPR). Early biosensors were based on PC-SPF [11], but GC-SPF has recently been applied to biosensors [15,16,17,18]. Wavelength-scale periodic structure coated with metal films is essential for GC-SPF method. We call it a plasmonic chip [15,16,17,18,19,20], which has been used as a sensor-chip. The plasmonic chip used with the SPF method has an overlayer composed of dielectric medium such as SiO_2_ on the metal film. The roles of the SiO_2_ layer are the protection of the metal layer from oxidation, suppression of a fluorescence quench by a metal layer [21], and the formation of an appropriate environment for biomaterial. The excited light can be incident from the top or bottom of a chip. The resonance condition is described as

***k*_spp_** = ***k*_ph_**·sin *θ* ± *m*·***k*_g_**, (3)

where ***k*_spp_**, ***k*_ph_**, and ***k*_g_** are the wavenumber vectors of the surface plasmon, the incident light, and the grating proportional to the inverse of pitch, respectively [22,23]. ***k*_spp_** was composed of complex dielectric constants for the metal and dielectric medium at the interface. *m* is an integer and *θ* is an incident angle or a detection angle (i.e., surface plasmon resonance angle for excitation and peak angle for surface plasmon coupled emission (SPCE), respectively) [24,25].

Immunosensors using GC-SPF have been applied to detect various biomarkers [15,16,17,26]. In the detection of α-fetoprotein, the plasmonic chip can quantitatively detect until 2 pg/mL, in which the illumination was incident from the top side of the chip. The detection limit was quite low, but it can be further improved if the fluorescence enhancement factor can increase by optimizing the structure of a plasmonic chip (i.e., silver-film thickness and SiO_2_-film thickness), maintaining the pitch size and groove depth. In this study, the interaction between Cy5 fluorescently-labeled streptavidin and biotin modified with the surface of a plasmonic chip was used to study the improvement of fluorescence enhancement on the plasmonic chip as the model system. The light source was a He-Ne laser with the wavelength of 632.8 nm, and emission with a wavelength of 670 nm was detected for Cy5 (Figure S1). The SPCE peak can be detected at the direction normal to surface, 0 degree, which is the resonance condition as denoted by Equation (3) at wavelength of 670 nm. The SPCE peaks obtained at ±*θ* can overlap at 0 degrees, and the single peak has a higher intensity than double peaks separated at ±*θ* degrees. The optimal plasmonic chip structure including duty ratio, groove depth, and shape was already studied in our previous papers [18,19]. In this study, the plasmonic structure of pitch and groove depth was kept, and the silver film thickness and the silica film thickness were studied in order to improve the fluorescence enhancement factor in the top illumination system.

## 2. Results

### 2.1. Surface Analysis

**Figure 1:**
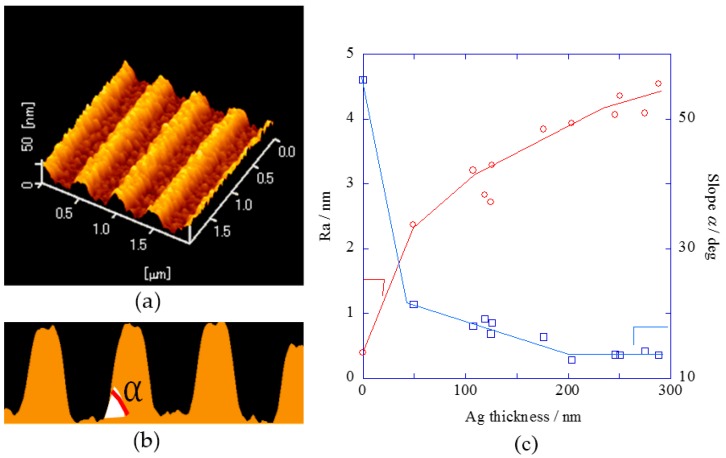
(**a**) Atomic force microscopy (AFM) image; (**b**) Cross-section contour of AFM image shows slope, α; and (**c**) Ra and slope α plotted against the silver film thickness.

Plasmonic chips with various silver film thickness or various SiO_2_ film thickness were fabricated, and they were measured by atomic force microscopy (AFM) (Figure S2). Cross-section contour of AFM images provided the slope α for a wall of convex in a periodic structure (Figure 1a,b), and surface roughness Ra was evaluated as the mean value of each Ra measured along the top of each convex line and the bottom of each groove line in AFM images (see Figure S2). Figure 1c shows the results of Ra and slope α plotted against the silver-film thickness. As the silver-film thickness increased, the roughness also gradually increased, and the slope was loose until silver film thickness of 200 nm, and then the slope kept constant over 200 nm.

### 2.2. SPF Measurement

#### 2.2.1. Silver-Film Thickness Dependence of the Fluorescence Intensity

Silver-film thickness dependence of fluorescence intensity was investigated for the two types of optical systems; the detection angles scanning under the incident angle were fixed to 45 degrees, corresponding to out-of-resonance angle (DAS) and the incident angles scanning under the detector were fixed to 60 degrees, corresponding to out-of-SPCE angle (IAS). From IAS, the fluorescence intensities excited at resonance angle (peak) and out-of-resonance angle (base) were evaluated. Similar to IAS, the fluorescence intensities detected at SPCE angle (peak) and out-of-SPCE angle (base) were evaluated in DAS.

**Figure 2:**
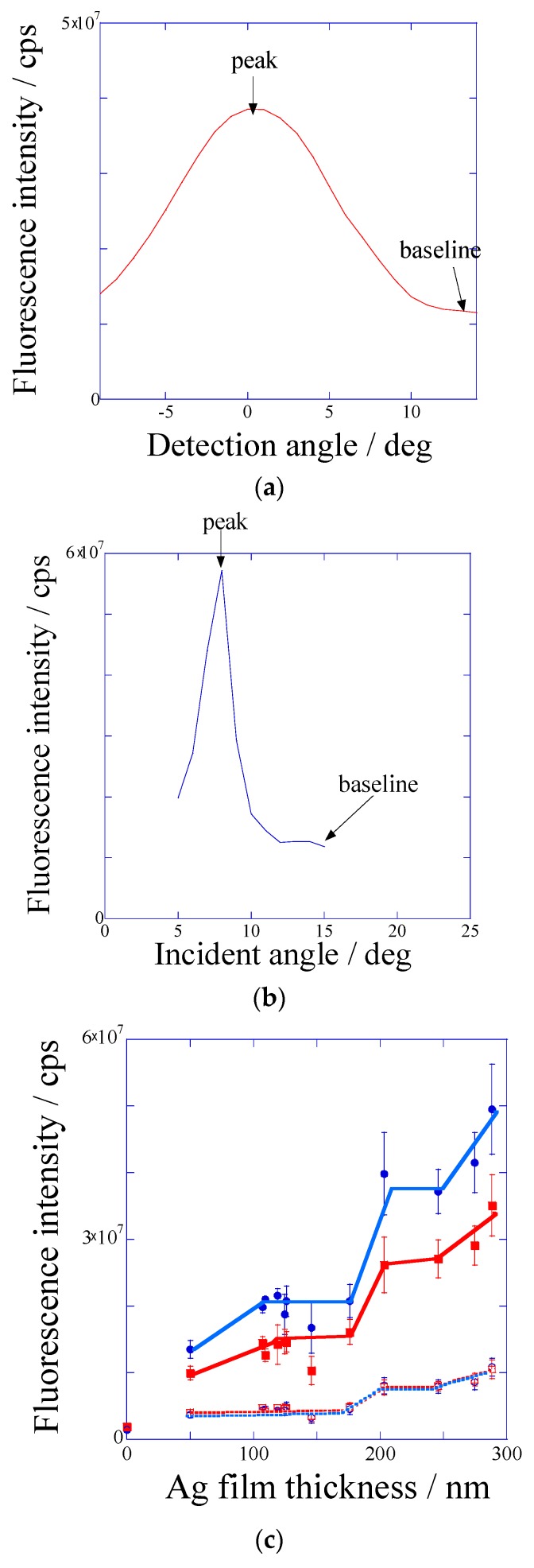
Fluorescence intensities measured (**a**) against the detection angle under the excitation at out-of-resonance angle (DAS) system and (**b**) against the incident angle under the detection at out-of-SPCE (surface plasmon coupled emission) angle (IAS) system; and (**c**) fluorescence intensities plotted against the silver film thickness for the peak values in DAS (red solid line) and IAS (blue solid line) and for the base values in DAS (red broken line) and IAS (blue broken line).

Figure 2c shows the fluorescence intensities for peaks and baselines in both DAS and IAS plotted against the silver-film thickness. The fluorescence intensities at base increased similarly to those at resonance peaks as the silver film became thick. Therefore, Figure 2 shows the silver-film thickness dependence of fluorescence enhancement by not only the plasmonic effect, but also the other optical effect. The surface roughness Ra—obtained by AFM images—also increased with the silver film thickness (as shown in Figure 1c), and the increase of fluorescence intensities was considered to be due to the increase of scattering effect based on the surface roughness.

**Figure 3:**
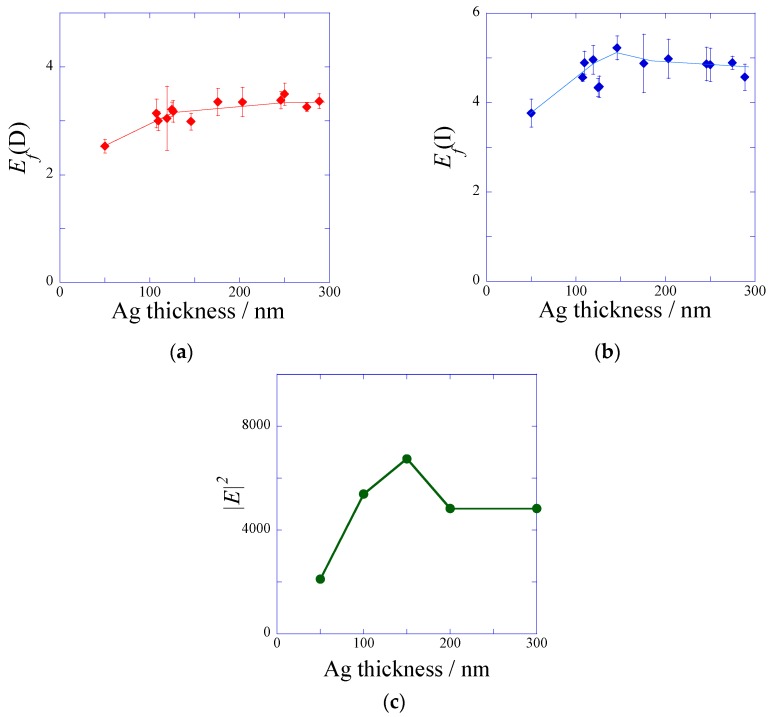
Enhancement factors against the silver film thickness in (**a**) DAS and (**b**) IAS measurement, which were evaluated as the fluorescence intensity at peak divided by the fluorescence intensity at baseline; and (**c**) the square of electric field intensity calculated against the silver film thickness by Finite-difference time-domain method (FDTD) method.

Plasmonic enhancement factors *E*_f_(D) and *E*_f_(I) were evaluated from the ratio of fluorescence intensity at peak to that at base in DAS and IAS systems in order to remove the scattering effect. As shown in Figure 3a, *E*_f_(D) for SPCE effect increased up to around 150 nm-thick silver film, and it kept constant above 150 nm. In the *E*_f_(I) for excited field enhancement shown in Figure 3b, the effect increased up to 150 nm and then it decreased over 150 nm. In both DAS and IAS, the increases of *E*_f_(D) and *E*_f_(I) for thinner silver film between 50 and 150 nm was considered to be due to suppressing the transmittance of illumination light by a silver film and improving the coupling efficiency between plasmon and light. On the other hand, the decrease of *E*_f_(I) for thicker silver film (more than 150 nm) was explained by the Finite-difference time-domain method (FDTD) simulation result (Figure 3c). The slope of wall for a convex in a periodic structure was loose at silver film thickness over 200 nm as shown in Figure 1c. In FDTD simulation, when the parameter of slope keeps constant, the electric field also keeps for silver film thickness of more than 150 nm. However, when the value of incline analyzed from AFM cross-section contour was applied to the calculation parameter in FDTD, the electric field steeply decreased at 150 nm-thick silver film. This is consistent with the decrease of *E*_f_(I).

The fluorescence intensity increased with silver-film thickness by the scattering effect due to the surface roughness. On the other hand, the coupling efficiency between incident light and surface plasmon, which is composed of suppressing the transmittance of incident light and the effect of incline of convex in periodic structure, were also considered to contribute to the fluorescence enhancement. Therefore, the surface selective fluorescence originating from GC-SPR was found to be maximized in 150 nm-thick silver film. The largest *E*_f_(D) and *E*_f_(I) values were found to be 3.2 and 5.1, respectively. The total plasmon effect of *E*_f_(D) × *E*_f_(I) was considered to be 16-fold. On the other hand, the fluorescence intensity was measured for the same assay on the glass slide as a reference. The mean fluorescence intensity on the glass slide was 1.8 × 10^6^ and 1.5 × 10^6^ in DAS and IAS, respectively. When the fluorescence intensities at base measured in DAS and IAS for 150 nm-thick silver film were divided by the mean fluorescence intensity on the glass slide, their values corresponded to the fluorescence enhancement factor by the reflection interference effect *E*_f_(R), and they were between 2.5-fold and 3-fold. Therefore, if the fluorescence excited at SPR angle was detected at SPCE peak, around 50-fold (*E*_f_(D) × *E*_f_(I) × *E*_f_(R) = 3.2 × 5.1 × 3) was obtained as the fluorescence enhancement *E*_f_.

#### 2.2.2. Dependence of the Fluorescence Intensity on the Distance from the Metal Surface

**Figure 4:**
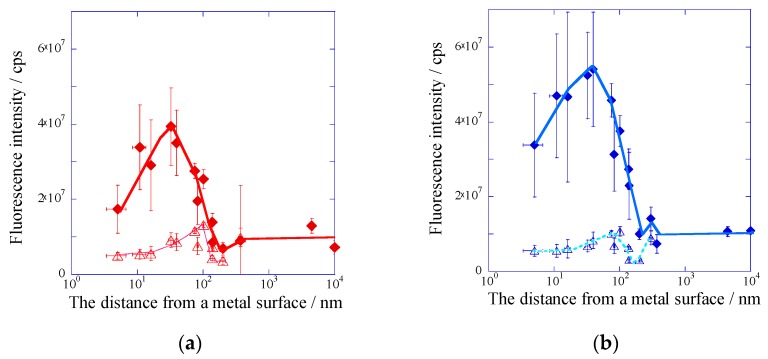
Fluorescence peak (solid line and rhombuses) and base (broken line and triangles) intensities plotted against the distance from a metal surface (**a**) in DAS system and (**b**) in IAS system.

The SiO_2_ overlayer of a plasmonic chip was prepared in various film thicknesses between 8–300 nm. The SiO_2_ film thickness was controlled by the sputtering time and the film thickness was evaluated with theoretical fitting of the reflectivity spectra measured by PC-SPR. The linearity between the sputtering time and SiO_2_ film thickness was confirmed previously. SiO_2_-film thickness corresponds to the distance from a metal surface, and the SiO_2_ layer suppresses fluorescence quench. The fluorescence curves were measured by DAS and IAS, and both fluorescence intensities for DAS and IAS evaluated under the resonance (peak) and off resonance (base) were plotted against the distance from a metal surface as shown in Figure 4a,b, respectively. In IAS and DAS, the most enhanced fluorescence was observed at the distance of 30–40 nm. The distance was larger than the values predicted from fluorescence quench to the silver film [27]. The enhanced fluorescence intensity includes three kinds of distance dependence; i.e., an exponential decay of an enhanced electric field by SPR, an excited energy transfer (=quench) to the metal surface by Förster energy transfer [28], and an electric field enhanced by the interference effect between incident light and reflection light. Except for the decay of an electric field, the quench effect and the interference effect were observed even for the flat metal surface or under off resonance. The distance dependences of the fluorescence intensities observed in the base (off resonance) in IAS and DAS were almost equivalent, and they were consistent with the square of the electric field intensity due to the reflection interference calculated by FDTD (Figure S3).

**Figure 5:**
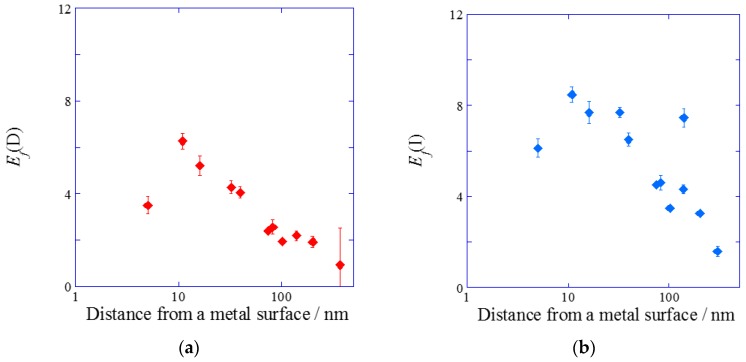
Enhancement factor obtained from the peak values divided by the base values (**a**) in DAS system and (**b**) in IAS system.

In a similar way to the metal film thickness dependence, the peak values were divided by the base values in DAS and IAS, individually, in order to remove the fluorescence quench and reflection interference effect by a metal film. The SiO_2_-film thickness dependences of *E*_f_(D) and *E*_f_(I) are shown in Figure 5a,b, respectively. In both cases, the largest fluorescence enhancement factor was observed at a distance of around 10 nm, and *E*_f_ decreased to 1 at several hundreds of nm. Their behaviors were based on the decay of the plasmon field. The fluorescence quench was known to disappear at 10 nm, which is the Förster radius between a thin silver film and a dye molecule discussed in the Chance-Prock-Silbey (CPS) model [27,28]. At SiO_2_ film thickness less than 10 nm, the fluorescence quench effect strongly depends on the distance from the metal surface, and it is very difficult to completely remove the quenching effect under 10 nm. The largest enhancement factors in DAS and IAS were found to be 6.5-fold and 8.7-fold at 10 nm from a silver surface (Figure 5a,b).

### 2.3. Limit of Detection for IAS and DAS

**Figure 6:**
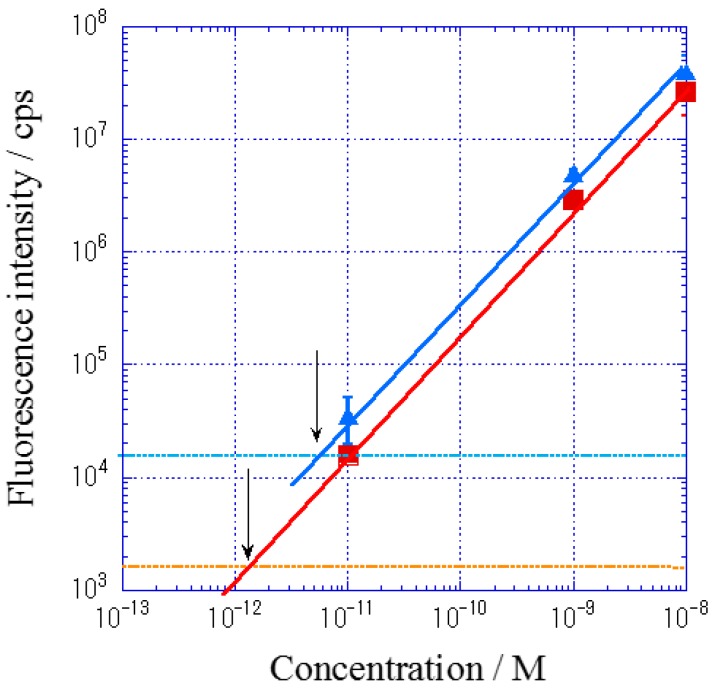
Calibration curve for Cy5-streptavidin (Cy5-SA): blue line shows the plots for IAS system and red line shows those for DAS system. Two broken lines with blue and red correspond to the standard deviation of background for IAS and DAS, respectively.

Figure 6 shows the calibration curve for Cy5-streptavidin (Cy5-SA). Fluorescence intensities were measured at three kinds of Cy5-SA concentration in DAS and IAS systems, and their mean values at peaks are plotted in Figure 6. The calibration curve in IAS is shown in the upper line in Figure 6, and each value was larger than that in DAS, depicted in the lower line. The standard deviation (SD) values in IAS were also larger than those in DAS, possibly due to the larger light scattering. The cross points of calibration curve and +SD line of background (as shown with arrows in Figure 6) indicate the limit of detection. Though the larger fluorescence intensity was observed in IAS, more sensitive detection was obtained in DAS.

## 3. Discussion

**Figure 7:**
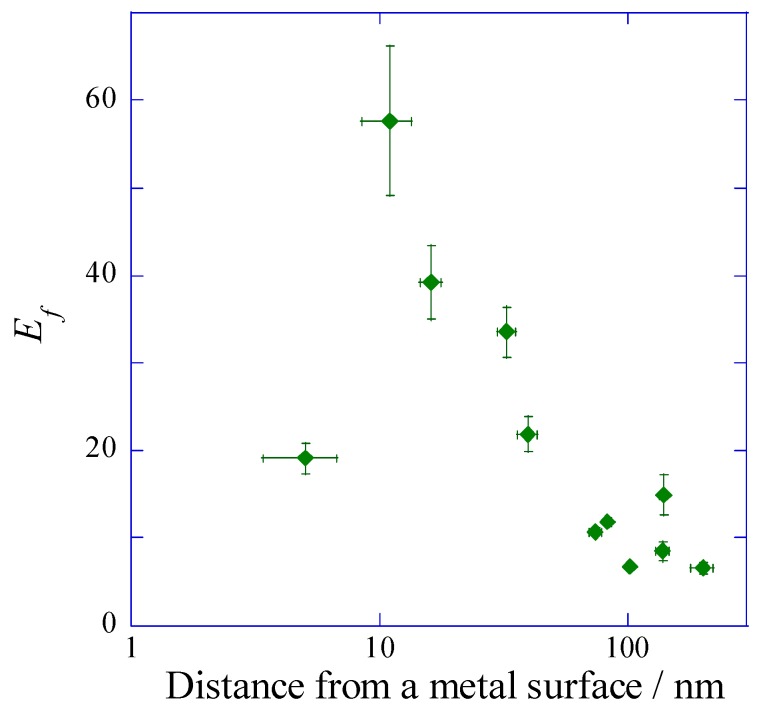
Fluorescence enhancement factor multiplied the normalized peak value to base value for DAS and that for IAS.

**Figure 8:**
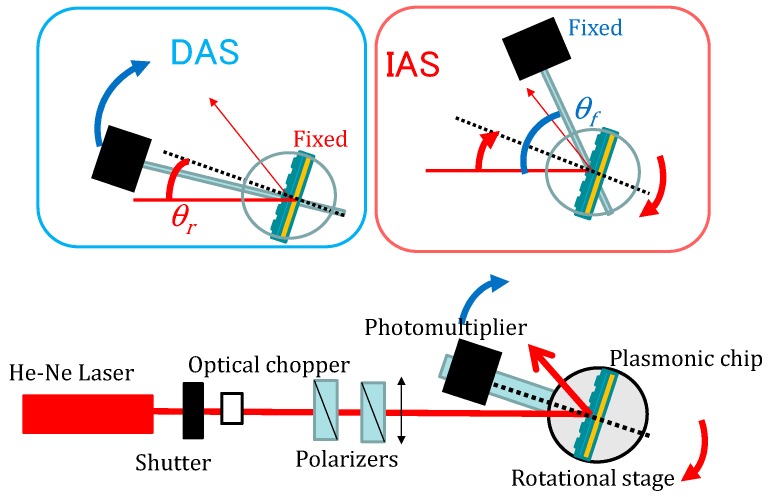
Surface plasmon field-enhanced fluorescence (SPF) setup and optical configuration for DAS and IAS measurement system.

The fluorescence enhancement factor *E*_f_ evaluated from the multiplication of *E*_f_(I) and *E*_f_(D) was plotted against the distance from a metal surface (Figure 7). The most enhanced fluorescence could be detected at the SPCE peak angle under the illumination at the incident angle of SPR. In this study, the resonance angle was 8 degrees for the plasmonic chip prepared with the silver-film thickness of 150 nm and the SiO_2_-film thickness of 10 nm. So, a detection angle of 0 degree means that the detector was set to 8 degrees against the incident beam line. In the configuration of the optical system, the detector was not set to 8 degrees, because the incident beam was blocked (Figure 8). The enhancement factor due to *E*_f_(I) and *E*_f_(D) reached 57-fold from calculation, as shown in Figure 7. When the plasmonic chip was used for immuno-detection, the fluorescence intensity measured included the three-fold reflection interference effect compared with the fluorescence intensity on the glass slide, and total enhancement reached around 170-fold (=57 × 3). The fluorescence enhancement factors have been reported in the other system. S. Bae et al. reported the values of 10–14 for silver nanowire based on the localized SPR [29], and Y. Wang et al. reported 100 times for fluorescence co-enhanced by localized SPR and propagating SPR [30]. Among the SPF systems, the enhancement factor of 170-fold based on the GC-SPR was the larger value. Higher sensitivity detection can be achieved by suppressing the background noise. As shown in Figure 6, comparing the limit of detection between IAS and DAS, IAS showed a larger fluorescence intensity than that in DAS and had larger standard deviation in background. As a result, the sensitive detection was observed in DAS, suppressing the standard deviation of background. Highly sensitive detection of biomarkers can be achieved by the largest fluorescence enhancement and the smallest deviation of background.

## 4. Materials and Methods

### 4.1. Fabrication of a Plasmonic Chip

The periodic structure of the plasmonic chip was fabricated by UV-nanoimprint method. The details were shown in a previous study [31]. The replica was coated with thin films of titanium silver, titanium, and silica in order. Titanium films were prepared with film thickness less than 1 nm. The other film thicknesses are summarized in Table 1. The pitch, groove depth, and duty ratio of the plasmonic chip were 480 nm, 27 nm, and 0.5, respectively.

Although the SPR angle was independent of the silver-film thickness, it shifted to higher angle as SiO_2_-film thickness increased (Figure S4).

### 4.2. Surface Measurement

The plasmonic chip was measured by AFM (SPI3800N, SII, Chiba, Japan). The mean surface roughness Ra was evaluated from roughness on the lines of convex part. The pitch and groove depth were also evaluated from AFM images. From cross-section contour, the slope of a wall for convex was evaluated.

### 4.3. Samples

All the plasmonic chips were modified with 3-aminopropyltriethoxysilane (APTES; Sigma-Aldrich, St. Louis, MO, USA) by silane-coupling reaction and biotin-polyethylene glycol-succinimide (NHS-PEGBio; BI-050TS, NOF, Tokyo, Japan) by chemical reaction with the amino group of APTES. Cover glass was attached to the sensor-chip with double-sided tape, and a gap was satisfied with solution by capillary phenomena. Cy5-SA (GE Healthcare, Little Chalfont, UK) prepared at 10 nM was applied to the fluorescence intensity measurement in order to optimize the plasmonic structure. For fluorescence measurement, the distance from the metal surface over 300 nm was prepared using 370 nm*φ*- or 4500 nm*φ*-microbeads as a binder and the cover glass modified with NHS-PEGBio was attached to the plasmonic chip. The Cy5-SA can be bound to biotin in the cover glass side. Furthermore, in order to make a comparison of the detection sensitivity between IAS and DAS systems, Cy5-SA was prepared at the concentrations of 10, 1 nM, and 10 pM, and their florescence intensity was measured by SPF setup.

### 4.4. Fluorescence Measurement

The measurement method is briefly shown here, and details were described in the previous papers [15,17,18]. A He-Ne laser beam of 632.8 nm wavelength with 1 mW passes through an optical chopper and two polarizers for intensity and polarization control. Using a *θ*-2*θ* goniometer, the light reflected on the substrate was monitored against an incident angle (*θ_i_*) of 5–30 degrees by a Si photodiode. The diameter of the illumination spot was about 1 mm. The emission was monitored by a photomultiplier tube (after passing through lenses and a narrow band interference filter (λ = 670 + 5 nm) and a notch filter (Stopline, NF03-633E, Semrock, New York, NY, USA) mounted on the goniometer unit instead of a Si photodiode. Incident angle *θ*_i_ and detection angle *θ*_f_ were defined as the angle between the laser beam line and normal to the chip and the angle between the direction of the detector and normal to the chip, respectively (Figure 8). For the incident light from the top of a panel, fluorescence intensity was measured against the detection angle between 10 and 15 or 50 degrees at the fixed incident angle of 45 degrees (DAS; left panel of upper in Figure 8) and that against the incident angle between 5 and 30 or 60 degrees at the fixed detection angle of 60 degrees (IAS; right panel of Figure 8, upper).

### 4.5. Numerical Analysis

The electric field near the surface of the grating patterns was calculated using the two-dimensional FDTD (FullWAVE, RSOFT, Synopsys Mountain View, CA, USA) method [32]. The calculation parameters for the grating models were as follows: the silica grating patterns had a pitch of 480 nm and a groove depth of 30 nm. The thickness of the silver layer was varied between 0–300 nm, and a 20 nm-thick SiO_2_ overlayer was prepared for the study of the silver film thickness-dependence of the fluorescence enhancement. Furthermore, the thickness of the SiO_2_ layer was varied between 10–300 nm, and a 150 nm-thick-silver layer was prepared for the study of the SiO_2_ film thickness-dependence of the fluorescence enhancement. A trapezoidal model was used as a surface profile. From the cross-sectional areas measured by AFM, the incline (slope) of a wall for convex was evaluated. The computational parameters of slope of a wall for convex were determined from cross-section contours for plasmonic chips fabricated as shown in Figure 1c. Calculations were executed for two periodically repeating units drawn using non-uniform grids ranging from 5 nm to 1 μm to reduce the computational time. The smallest grids of 5 nm were used for the regions close to the interface. A periodic boundary condition was used for the unit-cell edges in the direction orientated normal to the grating surface, and a perfectly matched layer absorbing boundary condition was used for the edges in the direction orientated parallel to the grating surface. The refractive index of SiO_2_ for an overlayer and a grating substrate, and of silver, was used as *n* = 1.457 and k = 0 and *n* = 0.015 and k = 4.13, respectively. The refractive index of water was used as *n* = 1.33. The FDTD computations were carried out in an incident plane with a wavelength of 632.8 nm and the polarization of transverse magnetic mode. The light source was placed in the matrix, and therefore, the incident angle of the propagating light into water was converted to the angle in air to adjust to the SPF experimental conditions.

## 5. Conclusions

A plasmonic chip can enhance the fluorescence intensity of fluorescent dye by the GC-SPF. The optimal structure of a sensor-chip was found to be covered with 150 nm-thick silver film and 10 nm-thick SiO_2_ film with a grating of 480 nm pitch under the irradiation from the top panel of a sensor chip. Enhancement factor is composed of *E*_f_(I) by the enhanced electric field (excitation field), *E*_f_(D) by the surface plasmon coupled emission (SPCE), and *E*_f_(R) by the reflection interference effect. The largest plasmonic enhancement calculated by *E*_f_(D) × *E*_f_(I) was 57-fold. The total enhancement factor adding reflection interference effect *E*_f_(R) was found to be around 170-fold. The enhanced fluorescence also induces a large deviation in background due to the scattering light. Therefore, not only the largest fluorescence intensity, but also the stable lower background noise were the most important factors for the sensitive detection.

## Figures and Tables

**Table 1:** Each film thickness prepared for each experiment.

Experiment	Silver/nm	SiO_2_/nm
Ag film thickness dependence	50–288	20 ± 4 (fixed)
SiO_2_ film thickness dependence	211 ± 8 (fixed)	8–300

## Supplementary Materials

The following are available online at www.mdpi.com/1996-1944/10/9/1063/s1. Figure S1: Cy5 absorption and fluorescence spectra from web page of GE healthcare. Figure S2: AFM images of plasmonic chips with various silver-film thickness and a top view of AFM image. Each silver-film thickness is: (a) 0 (replica before coating), (b) 49, (c) 126, (d) 175, (e) 203, (f) 245, (g) 250, (h) 275, (i) 288 nm, respectively. The surface roughness Ra was evaluated as the mean value of each Ra measured along the top of each convex line and the bottom of each groove line in AFM images as depicted with lines in (j). Figure S3: The square of the electric field intensity on the flat metal-coated substrate calculated against the distance from metal surface by FDTD method. Figure S4: (a) Reflectivity measured against the incident angle (SPR curves) for the plasmonic chip with SiO_2_- film thickness of 8 (red cross, ×), 16 (orange full circle, ●), 30 (green square, □), 41 (green full triangle, ▲), 72 (blue triangle, ▽), 140 (blue full square, ■), 200 (purple circle, ○), 300 (gray diamond, ◇) nm. (b) The SPR angle plotted against the SiO_2_-film thickness
