# Supplementary material for: Optimal Structure of a Plasmonic Chip for Sensitive Bio-Detection with the Grating-Coupled Surface Plasmon-Field Enhanced Fluorescence (GC-SPF)
Source: Materials (Basel). 2017 Sep 11;10(9):1063. doi: 10.3390/ma10091063 (PMC5615717; doi:10.3390/ma10091063)
Supplement: Supplementary file 1 [file materials-10-01063-s001.pdf]

Supplementary Materials: Optimal Structure of a Plasmonic Chip for Sensitive Bio-Detection with the Grating-Coupled Surface Plasmon-Field Enhanced Fluorescence (GC-SPF)

Keiko Tawa, Takuya Nakayama and Kenji Kintaka

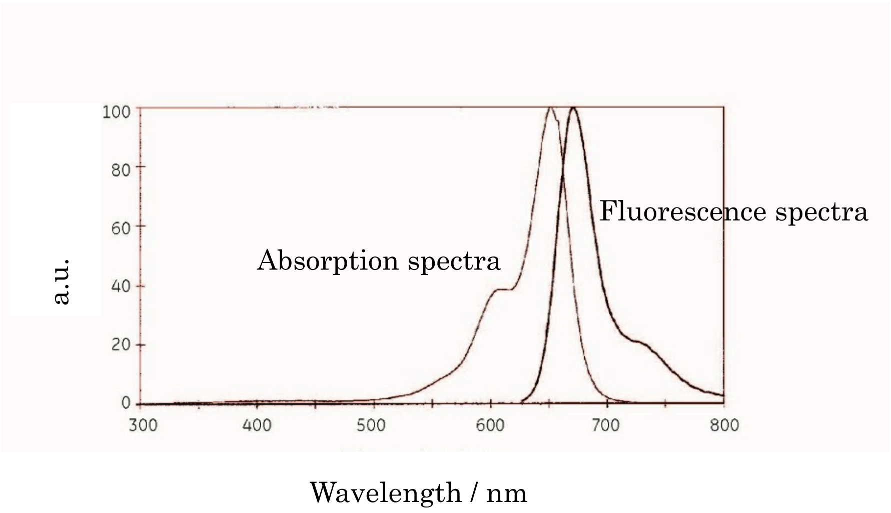

Figure S1. Cy5 absorption and fluorescence spectra from web page of GE healthcare. (https://www.gelifesciences.co.jp/catalog/0438.html)

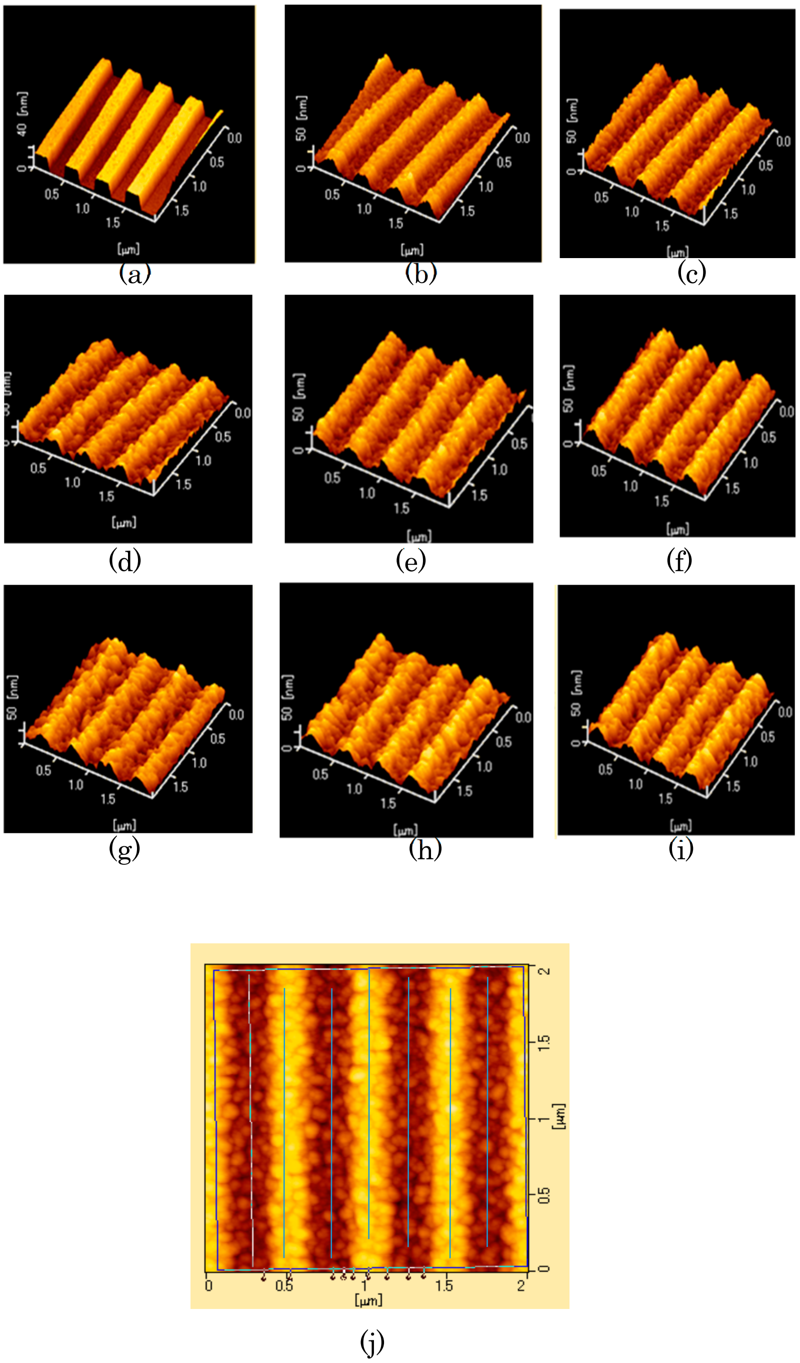

Figure S2. AFM images of plasmonic chips with various silver-film thickness and a top view of AFM image. Each silver-film thickness is: (a) 0 (replica before coating), (b) 49, (c) 126, (d) 175, (e) 203, (f) 245, (g) 250, (h) 275, (i) 288 nm, respectively. The surface roughness Ra was evaluated as the mean value of each Ra measured along the top of each convex line and the bottom of each groove line in AFM images as depicted with lines in (j).

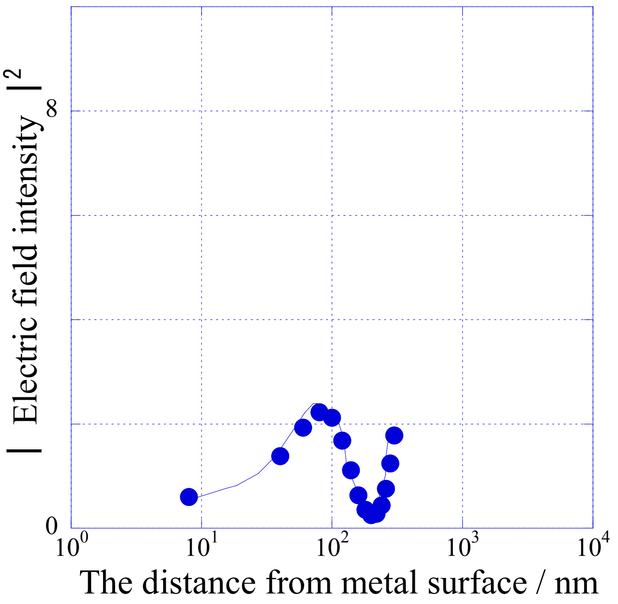

Figure S3. The square of the electric field intensity on the flat metal-coated substrate calculated against the distance from metal surface by FDTD method.

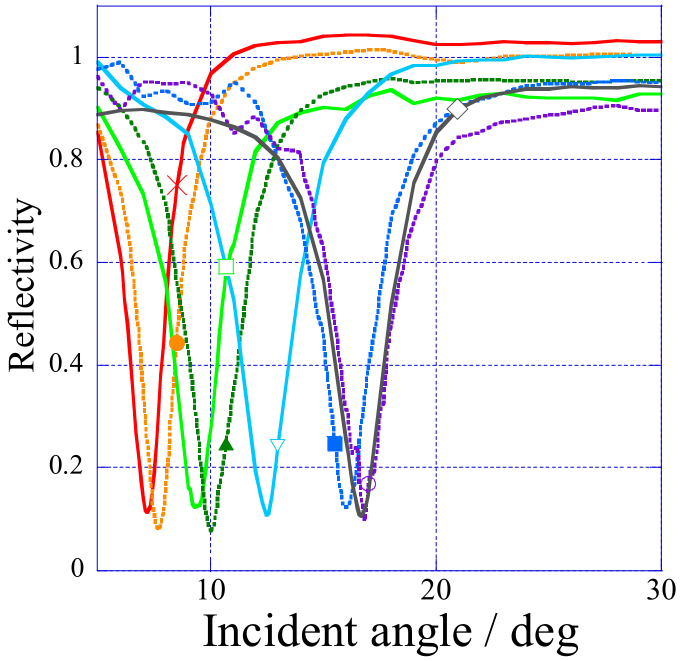

(**a**)

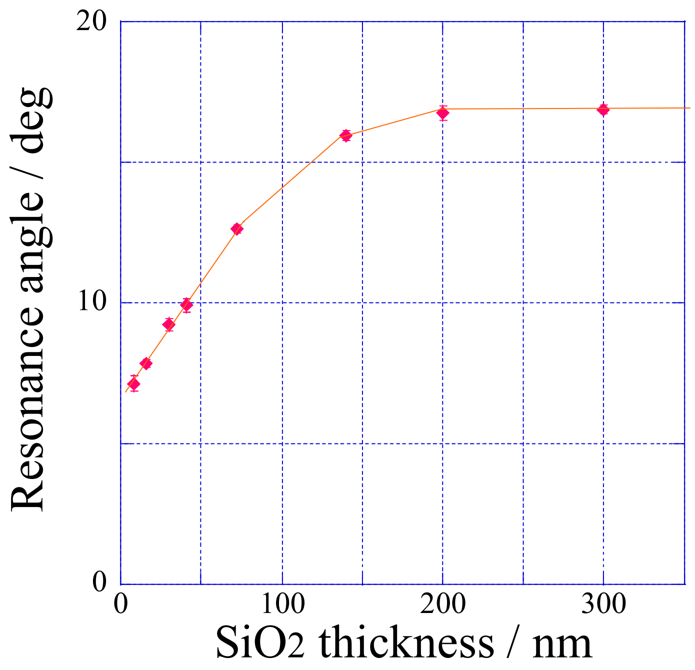

(**b**)

Figure S4. (**a**) Reflectivity measured against the incident angle (SPR curves) against SiO_2- film thickness of 8 (red cross, x), 16 (orange full circle, ●), 30 (green square, □), 41 (green full triangle, ▲), 72 (blue triangle, ▽), 140 (blue full square, ■), 200 (purple circle, ○), 300 (gray diamond, ◇) nm. (b) The SPR angle plotted against the SiO_2-film thickness.
